# Differences in characteristics between patients from Egypt and Germany presenting with lacunar stroke

**DOI:** 10.1038/s41598-023-50269-z

**Published:** 2023-12-21

**Authors:** Mohamed Maged, Hany Aref, Nevine El Nahas, Eman Hamid, Mai Fathy, Tamer Roushdy, Jan Hendrik Schaefer, Christian Foerch, Daniel Spitzer

**Affiliations:** 1 Department of Neurology, Ain Shams University, Cairo, Egypt; 2 Department of Neurology, Goethe University, Frankfurt, Germany; 3 Department of Neurology, Ludwigsburg Hospital, Ludwigsburg, Germany

**Keywords:** Stroke, Epidemiology

## Abstract

Despite the enormous health burden of lacunar stroke, data from low- and middle-income countries on lacunar stroke characteristics and its comparison with that of high-income countries are scarce. Thus, we aimed to investigate and compare the variable characteristics and vascular status in patients from Egypt and Germany suffering lacunar stroke. Two cohorts of lacunar stroke patients from Ain Shams University Hospital, Egypt and Goethe University Hospital Frankfurt, Germany were retrospectively collected between January 2019 and December 2020 and analyzed for demographics, risk factors, mode of presentation, neuroimaging features, treatment protocols and outcomes. MRI showed a different distribution pattern of lacunar strokes between cohorts, detecting posterior circulation lacunar infarctions preponderantly in patients from Egypt and anterior circulation lacunar infarctions preponderantly in patients from Germany. Complementary MR/CT angiography revealed a significantly higher proportion of intracranial and combined intracranial and extracranial arterial stenosis in patients from Egypt than in patients from Germany, suggesting differences in pathological processes. Younger age, higher NIHSS on admission, and posterior circulation lacunar infarction were predictors of Egyptian origin, whereas hypertension was a predictor of German origin. Our results support the idea of clinical and neuroimaging phenotype variations in lacunar stroke, including different sources of lacunar stroke in patients of different populations and geographical regions. This implies that guidelines for management of lacunar stroke might be tailored to these differences accordingly.

## Introduction

Cerebrovascular events have been recognized worldwide as the second cause of death and the third leading cause of disability. Around 15 million people suffer from a stroke annually, of whom one-third die and one-third remain with varying degrees of disability^1^. Lacunar strokes account for about 25% of all ischemic strokes^2^. They are associated with severe neurological deterioration in about one-third of lacunar stroke patients after symptom onset^3^ and development of cognitive dysfunction leading to high rates of long-term dependency^4^. Long-term consequences of lacunar stroke thus constitute an enormous health burden. The immense impact of lacunar stroke has led to a significant expansion of studies investigating prevalence, risk factors, pathophysiology and etiology of lacunar stroke within single geographically defined cohorts in high-income countries (HICs)^5^. It is worth mentioning that according to etiology of lacunar stroke, some of these studies indicated a role of stenotic lesions of large intracranial vessels in the development of lacunar syndromes associated with small deep infarctions on MRI in South Korean^6^ and Chinese patients^7^, whereas in Westerners atherosclerotic intracranial large artery stenosis was rather found as a coincidence than a causative of lacunar stroke^8^. Such a difference might arise from the fact that the prevalence of intracranial atherosclerosis is higher in Asian patients than in Westerners. One cohort study conducted in the low-middle income country Egypt also revealed a high prevalence of intracranial steno-occlusive arterial disease in ischemic stroke patients^9^ comparable with that of Asian patients. This study however was not specific to lacunar strokes but involved all ischemic stroke subtypes and did not compare Egyptian stroke patients to other populations^9^. According to this, data from low- and middle-income countries (LMICs) on lacunar stroke characteristics in which the prevalence of lacunar stroke is continuously increasing and perceived to be a major health burden^10^, are lacking. Thus, the paucity of information about lacunar stroke in several LMICs leads to a gap when it comes to comparing lacunar stroke characteristics among different populations and geographic regions.

Hence, we aimed to investigate clinical and neuroimaging characteristics in two different populations suffering lacunar stroke; one of them belongs to HICs (Germany) and the other to LMICs (Egypt). This study together with similar others might highlight the variance among lacunar stroke populations which can imply tailoring specific management guidelines accordingly.

## Methods

### Study design and patient selection

This is a retrospective study comparing two cohorts of patients from two countries, presenting with acute lacunar infarction. The two cohorts were compared for demographics, risk factors, mode of presentation, neuroimaging features, treatment protocols and outcomes.

We included 100 consecutive patients admitted to the stroke unit at the Ain Shams University Hospital, Cairo, Egypt and the Department of Neurology, Goethe University Hospital, Frankfurt, Germany between January 2019 and December 2020. Patients were diagnosed with magnetic resonance imaging (MRI)–verified lacunar stroke according to the STandards for ReportIng Vascular changes on nEuroimaging (STRIVE)^11^.

According to preset inclusion and exclusion criteria, stroke patients were retrospectively selected through a search of the hospitals’ databases with corresponding interfaces to laboratory and picture archiving and communication systems. Patients aged ≥ 45 years with an incident stroke were enrolled if brain MRI and MR or computed tomography (CT) angiography for detection of lacunar stroke and cranial arterial stenosis were conducted. Presence of intracranial hemorrhage, evidence of past or recent large territorial infarction, cerebral vasculitis, dissection and monogenic cause of stroke, and missing follow-up for modified Rankin Scale (mRS) 3 months after lacunar stroke led to exclusion of patients.

Importantly, the participating stroke units at Ain Shams University Hospital have been accredited as certified stroke centers by the LGA InterCert GmbH in concert with the German Stroke Society according to the German quality management system DIN ISO 9001 (Quality management systems—Requirements (ISO 9001:2015); German and English version EN ISO 9001:2015)^12^. Both the certified stroke units at Ain Shams University Hospital and the certified stroke center at Goethe University Hospital implemented the same stroke code protocols for individuals suspected of acute ischemic stroke (e.g., selection of patients in the prehospital phase of acute stroke for hospital admission and inhospital examinations, including selection of patients for MR imaging), thus assuring reliable comparability of analyzed data from an epidemiological standpoint.

The study fully adheres to the ethical principles of the Declaration of Helsinki as well as GCP guidelines and was approved by the local Ethics Committees at Ain Shams University Hospital (ethics approval number FMASU R91/2023) and Goethe University Hospital (ethics approval number 2021-21). Due to the retrospective nature of the study, the need of informed consent was waived by the Medicine Research Ethics Committee at Ain Shams University Hospital and the Medicine Ethics Commission at Goethe University Hospital.

### Definition of lacunar stroke and imaging analysis

Lacunar stroke was defined according to the criteria of the Trial of Org 10,172 in Acute Stroke Treatment (TOAST) classification system^13^ as presenting clinically with new onset of neurological deficit lasting ≥ 24 h and occurring in association with a focal hyperintense subcortical or brain stem lesion with a diameter ≤ 15 mm detected on diffusion weighted imaging (DWI) that would explain the symptoms^11^.

Cranial arterial stenosis was defined as ≥ 50% luminal stenosis according to standard methods^14,15^ in any of the major extracranial and/or intracranial arteries in MR or CT angiography: extracranial carotid arteries (ECA), extracranial vertebral arteries (EVA), intracranial internal carotid arteries (ICA), middle cerebral arteries (MCA), anterior cerebral arteries (ACA), posterior cerebral arteries (PCA), intracranial vertebral arteries (VA) and basilar artery (BA). Radiological analysis was performed independently by two expert neurologists (EHA and MFA in Egyptian sample, CF and DS in German sample), and in case of disagreement a consensus was reached.

### Assessment of patient characteristics

Demographic and medical information of enrolled lacunar stroke patients were obtained from medical records, including age, sex, cerebrovascular risk factors and plasma levels of lipids and hemoglobin A1c (HbA1c), medication given on admission, stroke severity as measured by the National Institutes of Health Stroke Scale (NIHSS) on admission and discharge, and mRS at discharge and 3 months after lacunar stroke, acute management and early secondary stroke prevention protocols.

### Statistical analysis

Descriptive statistics of lacunar stroke patients’ clinical characteristics and frequency of cranial arterial stenosis are summarized by nationality of included patients and presented using numbers for categorical variables, and the mean and standard deviation for continuous variables. For intergroup comparison χ^2^ test, Fisher’s exact test, and Mann–Whitney U test were used as appropriate. Subsequently, a logistic regression model was constructed with odds ratio (OR) and 95% confidence interval (CI) to identify clinical factors predicting nationality of lacunar stroke patients. All analyses were performed using SPSS for Windows version 19.0 (SPSS Inc., Chicago, Illinois) and Graph Pad Prism 9.0 (Graph Pad Software Inc., La Jolla, California, USA). A *P* value < 0.05 was considered statistically significant.

## Results

A total of 100 patients from Germany and 100 patients from Egypt presenting with lacunar infarction were included in the study. 100% of patients from Germany and Egypt had follow-up 3 months after onset of lacunar stroke, respectively. The baseline characteristics and risk factors of both groups are shown in Table 1. Compared to patients from Germany, patients from Egypt with lacunar stroke were significantly younger (mean ± SD age, 63.6 ± 9.2 years vs. 67.9 ± 12.7 years; *P* < 0.05) and had more severe neurological symptoms on admission (median NIHSS 5 vs. 3, *P* < 0.0001) and at discharge (median NIHSS 3 vs. 1, *P* < 0.0001). Relevant underlying cardiovascular risk factors and disability in activities of daily living were observed among both nationalities but with significantly higher rates of diabetes mellitus (n = 55 vs. 41,* P* < 0.05) and elevated HbA1c (mean ± SD, 7.1 ± 1.6 vs. 6.2 ± 1.3, *P* < 0.0001) in patients from Egypt, which, consequently, was associated with a more frequent antidiabetic therapy pre-admission (n = 54 vs. 31, *P* < 0.01). Furthermore, higher rates of history of cigarette smoking (n = 38 vs. 24, *P* < 0.05), chronic heart disease (n = 23 vs. 5, *P* < 0.001) and in trend previous stroke or transient ischemic attack (n = 26 vs. 16, *P* = 0.08) were observed among the Egyptian cohort. In patients from Germany, on the other hand, we detected a higher comorbidity of hypertension (n = 85 vs. 63, *P* < 0.001) and dyslipidemia (n = 56 vs. 23, *P* < 0.0001) that was associated with more frequent intake of antihypertensive and lipid-lowering agents, and higher levels of disability (median mRS 1 vs. 0, *P* < 0.05). With respect to therapeutic management of lacunar stroke, rt-PA and dual loading dose of antiplatelets were more frequently administered to patients from Egypt compared to patients from Germany on admission (rt-PA administration, n = 16 vs. 8, P = 0.08; administration of loading antiplatelets, n = 62 vs. 4, P < 0.0001). The preferred secondary stroke prevention strategies were mono antiplatelet therapy in patients from Germany (n = 79 vs. 0, P < 0.0001) and dual antiplatelet therapy in patients from Egypt (n = 93 vs. 10, P < 0.0001).

**Table 1:** Baseline characteristics of patients with lacunar stroke.

	Egyptian cohort *N* = 100	German cohort *N* = 100	*P* value
Patient-related factors			
Age, years	63.6 ± 9.2	67.9 ± 12.7	< 0.05
Sex			
Male	71	62	0.1776
Female	29	38	
Risk factors			
Current smoker	38	24	< 0.05
Hypertension	63	84	< 0.001
Diabetes mellitus	55	41	< 0.05
HbA1c (%)	7.1 ± 1.6	6.2 ± 1.3	< 0.0001
Dyslipidemia	23	56	< 0.0001
Total cholesterol (mg/dl)	190.9 ± 55.4	184.8 ± 55.9	0.3196
LDL-C (mg/dl)	118.0 ± 43.5	112.5 ± 47.1	0.2573
HDL-C (mg/dl)	37.3 ± 9.5	47.5 ± 16.7	< 0.0001
Triglycerides (mg/dl)	160.5 ± 89.1	156.1 ± 97.4	0.5623
Atrial fibrillation	5	11	0.1179
Previous myocardial infarction	1	3	0.6212
Chronic heart disease	23	5	< 0.001
Previous stroke/TIA	26	16	0.0826
Medication use on admission			
Antihypertensive agent	43	63	< 0.01
Antidiabetic agent or insulin	54	31	< 0.01
Lipid-lowering agent	15	27	< 0.05
Antiplatelet agent	27	32	0.4382
Anticoagulant	2	9	0.0582
Clinical scores			
NIHSS on admission	5 (3–7)	3 (1–5)	< 0.0001
NIHSS at discharge	3 (2–5)	1 (0–4)	< 0.0001
mRS before admission	0 (0–0)	0 (0–1)	< 0.0001
mRS at discharge	1 (1–2)	1 (0–3)	0.8308
mRS after 3 months	0 (0–1)	1 (0–2)	< 0.05
Acute treatment			
Actilyse (rt-PA)	16	8	0.0817
Thrombectomy	0	2	0.1552
Loading of antiplatelets	62	4	< 0.0001
Early secondary stroke prevention			
Mono antiplatelet therapy	0	79	< 0.0001
Dual antiplatelet therapy	93	10	< 0.0001
Oral anticoagulation	7	11	0.3230

Values are presented as numbers, mean ± SD or median (IQR).

*LDL-C* indicates low density lipoprotein cholesterol, *HDL-C* high density lipoprotein cholesterol, *TIA* transient ischemic attack, *NIHSS* National Institutes of Health Stroke Scale, and *mRS* modified Rankin Scale.

**Figure 1:**
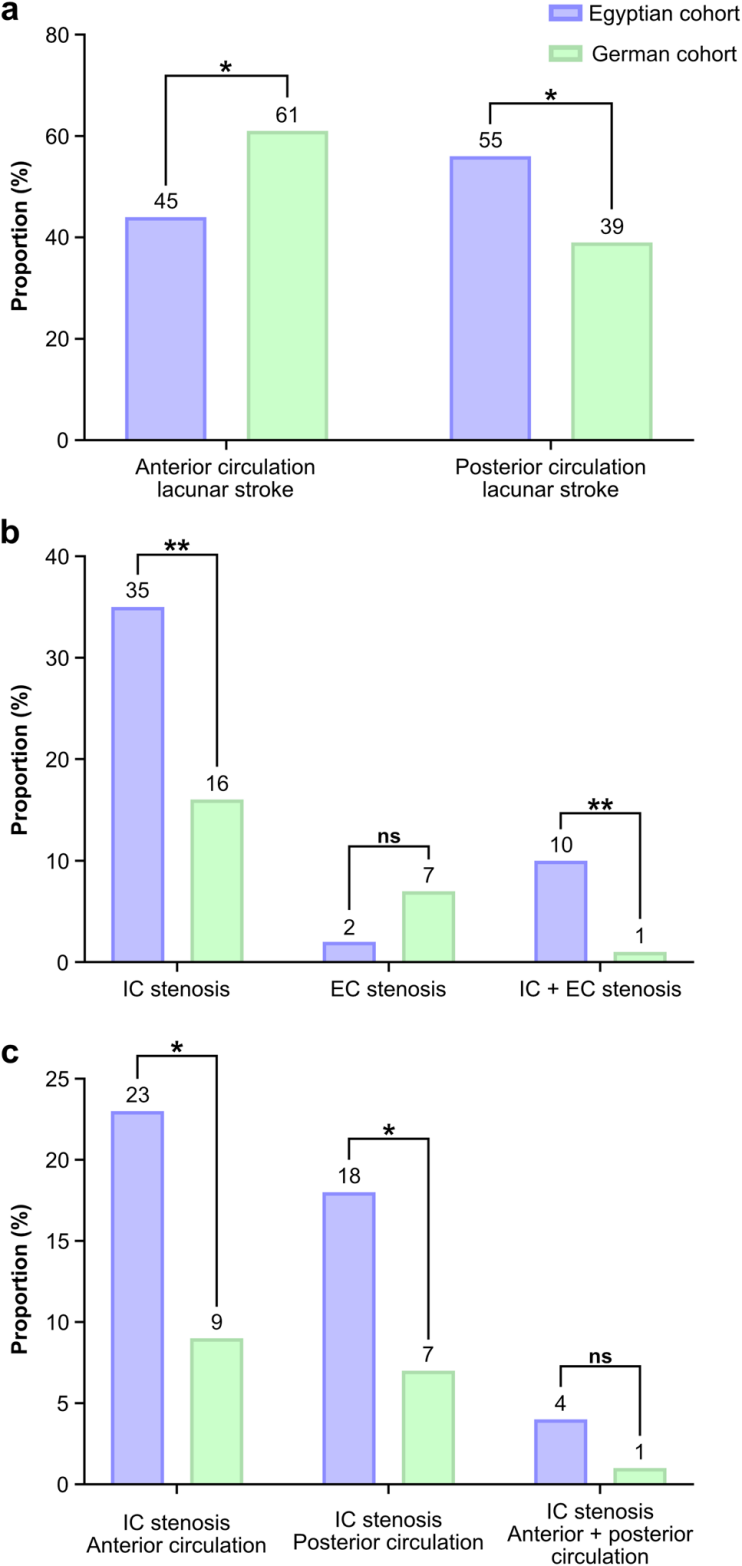
Distribution of lacunar strokes and proportions of intracranial and extracranial arterial stenosis in patients from Egypt and Germany. (**a**) Proportion of anterior and posterior lacunar infarctions, (**b**) proportion of intracranial and extracranial stenosis and (**c**) proportion of intracranial anterior and posterior circulation stenosis. *IC* intracranial, *EC* extracranial.

Diffusion weighted imaging (DWI) revealed a different distribution pattern of lacunar stroke between cohorts, more frequently detecting posterior circulation lacunar stroke in patients from Egypt (n = 55 vs. 39, *P* < 0.05) and anterior circulation lacunar stroke in patients from Germany (n = 61 vs. 45, *P* < 0.05) (Fig. 1a). In patients from Egypt, anterior lacunar strokes were predominantly detected in the basal ganglia (n = 13), internal capsule (n = 12) and corona radiata (n = 11), and posterior lacunar infarctions in the brainstem (n = 35) and thalamus (n = 15). In patients from Germany, anterior lacunar strokes were mainly found in basal ganglia (n = 24), internal capsule (n = 16) and deep white matter (n = 10), and posterior lacunar strokes in the brainstem (n = 29) and thalamus (n = 9) (Supplementary Table 1). Comparing the frequency of lacunar strokes in the above-mentioned anatomic regions among patient cohorts, DWI revealed significantly higher rates of lacunar stroke located in the basal ganglia in patients from Germany than in patients from Egypt, whereas frequency of lacunar strokes in other anatomic regions did not differ significantly.

The proportion of patients with either isolated intracranial (IC) arterial stenosis or combined IC and extracranial (EC) arterial stenosis was significantly higher in the Egyptian study population than in the German study population (IC arterial stenosis, n = 35 vs. 16, *P* < 0.01; IC + EC arterial stenosis, n = 10 vs. 1, *P* < 0.01; Fig. 1b), whereas the number of patients from Germany with isolated EC arterial stenosis exceeded the number of patients from Egypt. The latter however was not statistically significant (n = 7 vs. 2, *P* > 0.05; Fig. 1b). The intracranial anterior and posterior circulation were similarly affected by stenosis both in the Egyptian (anterior IC arterial stenosis, n = 23 and posterior IC arterial stenosis, n = 18) and German cohort (anterior IC arterial stenosis, n = 9 and posterior IC arterial stenosis, n = 7). The number of patients from Egypt with anterior and posterior circulation stenosis, however, significantly exceeded that of patients from Germany respectively (anterior IC arterial stenosis, n = 23 vs. 9, *P* < 0.05; posterior IC arterial stenosis, n = 18 vs. 7, *P* < 0.05; Fig. 1c). The number of patients with combined anterior and posterior circulation stenosis was low both in the Egyptian and German study population and did not differ significantly among cohorts (N = 4 vs. 1, *P* > 0.05, Fig. 1c).

In multivariate regressions as shown in Table 2, younger age (odds ratio [OR] 0.95, 95% CI [0.93–0.98], *P* < 0.01), higher NIHSS on admission (odds ratio [OR] 1.22, 95% CI [1.10–1.36], *P* < 0.001) and posterior circulation lacunar infarction (odds ratio [OR] 2.27, 95% CI [1.20–4.31], *P* < 0.05) were predictors of Egyptian origin, whereas hypertension (odds ratio [OR] 0.24, 95% CI [0.11–0.52], *P* < 0.001) was a predictor of German origin.

**Table 2:** Multivariate regression analysis: variables predicting Egyptian (vs. German) nationality of patients with lacunar stroke.

	OR	95% CI	*P* value
Gender	1.19	0.06–2.35	0.616
Age	0.95	0.93–0.98	< 0.01
Hypertension	0.24	0.11–0.52	< 0.001
Diabetes mellitus	1.89	0.98–3.64	0.054
NIHSS on admission	1.22	1.10–1.36	< 0.001
Posterior circulation lacunar infarction	2.27	1.20–4.31	< 0.05

*OR* odds ratio, *CI* confidence interval.

## Discussion

Lacunar stroke is responsible for one-quarter of the overall number of ischemic strokes with long-term complications, therefore carrying health and economic issues for patients and health care systems^4^. This particularly applies for LMICs, where the prevalence of lacunar strokes reaches 45% to 60% in some regions of the Middle East^16,17^, which is relatively higher than in HICs^18–20^. Despite the immense health burden of lacunar stroke in LMICs^10^, lacunar stroke characteristics have been poorly investigated in these countries compared to HICs, leading to an imbalanced representation of information on lacunar stroke in the published literature^5^. Hence, investigation of lacunar stroke characteristics and its disparities among different global regions would add great value to the overall understanding and management of lacunar stroke, which is a common and heterogeneous disease that necessitates a nuanced approach to diagnosis and treatment.

This comparative retrospective study is the first to explore clinical and neuroimaging characteristics of lacunar stroke and its disparities between patients from Egypt and Germany. We found a younger age at onset of lacunar stroke in patients from Egypt and a different anatomical distribution of lacunar stroke on MRI, more frequently detecting posterior circulation lacunar stroke in patients from Egypt and anterior circulation lacunar stroke in patients from Germany. These results presented here are in line with findings from previous comparative multiethnic or biethnic population studies^21–23^. Benavente and colleagues reported a higher incidence of lacunar infarctions in the brainstem or cerebellum in Black patients and Hispanics compared with non-Hispanic White patients^21^. Tsai et al.^22^ and Shu et al.^23^ identified Asian patients to have a younger age-onset of preponderant posterior circulation lacunar stroke compared to White patients with older age-onset of preponderant anterior circulation lacunar stroke. In addition, brain imaging revealed a high susceptibility to high-grade (> 50%) intracranial atherosclerotic arterial stenosis in patients from Egypt with more than double the rate compared to patients from Germany, affecting both the anterior and posterior circulation. The number of high-grade extracranial atherosclerotic arterial stenosis was very low among all lacunar stroke patients and did not differ between cohorts. The differences in frequency of intracranial atherosclerotic arterial stenosis between patients from Egypt and Germany presented here are well reflected by data of prospective clinical studies observing a high prevalence of intracranial atherosclerotic arterial stenosis in Egyptian patients^9^, and in patients of Asian ancestry, Hispanics and Africans^24^.

The high occurrence of high-grade intracranial atherosclerotic arterial stenosis detected in the Egyptian study population led to modification of the local guidelines for therapeutic management of lacunar stroke patients at the participating stroke units at Ain Shams University Hospital and is the cause of the deviating therapeutic management of lacunar stroke in patients from Egypt and Germany in the current study.

The observed disparities in neuroimaging findings might suggest different scenarios of pathophysiological processes involved in patients from Egypt and Germany presenting with lacunar stroke. Indeed, lacunar strokes have been reported to be attributable to disease of penetrating branches of large cerebral arteries in the absence of any disease of the parent artery, representing cerebral small vessel diseases, or atheromatous disease of the parent artery involving the origin of the penetrating branches further resulting in lacunar infarction^25^. Given that in almost half of patients from Egypt intracranial atherosclerotic arterial stenosis was detected, it allows to speculate about large artery atherosclerosis as another source of lacunar infarction, whereas very low rates of intracranial atherosclerotic arterial stenosis in patients from Germany might indicate cerebral small vessel diseases as the primary source of lacunar stroke. However, further prospective investigations with larger sample size are needed to distinguish between these different pathophysiological scenarios.

Factors probably contributing to different sources of lacunar stroke in patients of different ethnicity might be broad in spectrum, including complex interactions between genetic, biological and socioeconomic variables when compared to Whites. The role of genetic background of non-White patients, novel genetic variants, or genetic susceptibility compared to White patients and its contribution to lacunar stroke, intracranial atherosclerosis, as well as the risk factors associated with intracranial atherosclerosis are still incompletely understood and beyond the scope of this article. However, we analyzed several biological/clinical factors that have been shown to be associated with intracranial atherosclerosis and lacunar stroke risk^26^. To date, age and hypertension are the most recognized risk factors of lacunar stroke attributable to cerebral small vessel diseases, while others such as diabetes mellitus and smoking are still controversially discussed, possibly reflecting different underlying pathogenesis of lacunar stroke (e.g., intracranial atherosclerosis)^27,28^. We detected significantly higher rates of history of cigarette smoking, chronic heart disease, diabetes mellitus, and increased measured HbA1c in patients from Egypt, whereas hypertension and dyslipidemia were found to be more prevalent in patients from Germany. These differences in risk factor profiles are well reflected by the higher proportion of antidiabetic agent and insulin use in the Egyptian population as compared to German population offset by a greater proportion of antihypertensive and lipid-lowering agent use in the German population.

Moreover, according to NIHSS on admission and discharge, neurological deficits were significantly more severe in patients from Egypt with qualifying lacunar stroke, whereas patients from Germany suffered from an increased disability three months after lacunar infarction. The more severe impairment in patients from Egypt with lacunar stroke on admission and discharge compared to patients from Germany might probably be attributable to the presence of underlying more severe stenotic lesions of intracranial vessels, which was also reported for patients with ischemic lacunar stroke and intracranial steno-occlusive arterial disease^29^. The significantly increased mRS in patients from Germany in our study might be an age-dependent effect as older age in lacunar stroke patients was shown to be associated with worsening long-term disability, even without recurrence^30^.

The paucity of presented data regarding lacunar stroke does not allow us to compare our populations to other major lacunar stroke populations. However, when comparing the Egyptian and German phenotype of lacunar stroke to those of others investigated in large epidemiological stroke studies^23,26^ there are some striking similarities. Palacio and coworkers identified that patients with diabetes mellitus and recent lacunar stroke participating in the Secondary Prevention of Small Subcortical Strokes (SPS3) randomized trial were significantly younger and more likely to be non-White patients with almost double the rates of intracranial arterial stenosis^26^. In contrast, Shu and colleagues reported a higher proportion of hypertension and an increased age in White patients with cerebral small vessel disease and absence of large cerebral artery stenosis^23^, further supporting the idea of clinical and brain imaging phenotype differences, including different sources of lacunar stroke in patients of different populations and geographical regions.

It is of note that the detected clinical and neuroimaging phenotype differences between patients from Egypt and Germany with lacunar stroke are unlikely to be biased (e.g., selection bias of consecutively recruited patients with lacunar stroke and classification bias of lacunar stroke subtype); first, the participating Egyptian and German stroke units are both accredited as certified stroke centers according to the German quality management system using the same stroke code protocols for individuals suspected of acute ischemic stroke and second, both patients from Egypt and Germany were diagnosed with magnetic resonance imaging (MRI)–verified lacunar stroke according to the STandards for ReportIng Vascular changes on nEuroimaging (STRIVE)^11^, thus assuring reliable comparability of analyzed data.

The study, however, was not without limitations. The tremendously high number of lacunar stroke patients with a high susceptibility to high-grade (> 50%) intracranial atherosclerotic arterial stenosis detected in the Egyptian study population led to modification of the local guidelines for therapeutic management of lacunar stroke patients at the participating stroke units at Ain Shams University Hospital. Thus, the deviating therapeutic management of lacunar stroke in patients from Egypt and Germany may have resulted in different outcomes among the two study populations. With respect to NIHSS scores of stroke patients depicted by Nahas and colleagues in a previously published multicenter retrospective cross-sectional study^31^ exploring sex differences in risk factors, stroke severity, quality of service and stroke outcome in 4620 patients from Egypt, both patients with minor and major stroke are admitted to the stroke center at Ain Shams University Hospital. However, due to the nature of the study protocol, we were unable to provide the Median and interquartile range of NIHSS scores in a larger cohort of stroke patients to better depict the ratio of patients with minor and major stroke symptoms in the Egyptian study population. Finally, analysis of differences in time from onset of stroke symptoms to presentation in the emergency department and frequency of clinical lacunar syndromes between patients from Egypt and Germany was not part of the study protocol. Thus, our study lacked a more detailed investigation of differences in acute stroke treatment processes and clinical presentation of lacunar stroke between patients from Egypt and Germany.

## Conclusions

Our findings suggest that lacunar strokes are associated with higher rates of intracranial arterial stenosis in patients from Egypt and might have a distinct pathophysiology compared with patients from Germany. The differences in clinical and brain imaging phenotype detected between patients from Egypt and Germany in our study further support the idea that lacunar stroke might not only be attributable to a pathophysiologic process (e.g., lipohyalinosis) in an adjacent penetrating artery but also to atheromatous disease of the parent artery involving the origin of the penetrating branches. Consequently, patients from Egypt with lacunar strokes should be evaluated for large artery disease before ascribing them to isolated small penetrating artery disease. However, these observed differences need to be confirmed and clarified by further prospective studies. Such studies might also include characterization of clinical and brain imaging phenotype differences in younger patients of different populations and geographical regions to obtain a better knowledge of the full clinical expression of this ischemic stroke subtype.

### Supplementary Information

Supplementary Table 1.

## Data Availability

The data supporting the findings of this study are available from the corresponding author upon reasonable request.
