# Supplementary material for: Differences in characteristics between patients from Egypt and Germany presenting with lacunar stroke
Source: Sci Rep. 2023 Dec 21;13:22925. doi: 10.1038/s41598-023-50269-z (PMC10739735; doi:10.1038/s41598-023-50269-z)
Supplement: Supplementary file 1 — Supplementary Table 1. [file 41598_2023_50269_MOESM1_ESM.pdf]

Differences in characteristics between patients from Egypt and Germany presenting with lacunar stroke

Mohamed Maged[1], Hany Aref[1], Nevine El Nahas[1], Eman Hamid[1], Mai Fathy[1], Tamer Roushdy[1], Jan Hendrik Schaefer[2], Christian Foerch[2,3], Daniel Spitzer[2]

[1]Department of Neurology, Ain Shams University, Cairo, Egypt

[2]Department of Neurology, Goethe University, Frankfurt, Germany

[3]Department of Neurology, Ludwigsburg Hospital, Ludwigsburg, Germany

Correspondence to:

Dr. Daniel Spitzer, Department of Neurology, Goethe University, Schleusenweg 2-16, 60528 Frankfurt, Germany, Tel.: +49-152-31836428, Email: Spitzer@med.uni-frankfurt.de

	Egyptian cohort *N*=100	German cohort *N*=100	*P* value
**Localization of lacunar stroke**			
Anterior circulation	45	61	<0.05
Basal ganglia	13	24	<0.05
Internal capsule	12	16	0.4150
External capsule	0	1	>0.9999
Corona radiata	11	5	0.1179
Centrum semiovale	2	3	>0.9999
Periventricular WM	2	2	>0.9999
Deep WM	5	10	0.1795
Posterior circulation	55	39	<0.05
Thalamus	15	9	0.1917
Cerebellum	5	1	0.2116
Brainstem	35	29	0.3631
**Fazekas score**			
Fazekas 0	16	10	0.2071
Fazekas 1	43	47	0.5697
Fazekas 2	24	30	0.3393
Fazekas 3	17	13	0.4283
**Cerebral microbleed**	12	10	0.6513

Supplementary Table 1. Comparison of neuroimaging features in patients from Egypt and Germany presenting with lacunar stroke. Values are given as numbers. WM indicates white matter; PVH, periventricular hyperintensities; DWMH, deep white matter hyperintensities; IWMH, infratentorial white matter hyperintensities.
